# Coefficient bounds for starlike functions involving *q*− Hurwitz-Lerch Zeta operator in conic region

**DOI:** 10.1016/j.heliyon.2024.e33403

**Published:** 2024-06-27

**Authors:** K. Uma, K. Vijaya

**Affiliations:** Department of Mathematics, School of Advanced Sciences, Vellore Institute of Technology, Deemed to be University, Vellore, Tamilnadu, India

**Keywords:** Symmetric conic domain, *q*-Hurwitz-Lerch Zeta function, Holomorphic functions, *q*-difference operator, Subordination results, Partial sums

## Abstract

In this paper, we generalize a family of *q*-Hurwitz-Lerch Zeta function by means of constructing and investigating a new family of analytic functions. Some novel findings are discussed like contraction coefficient inequality and other important concepts, some of which are: partial sums, coefficient estimates, subordination results for Janowski starlike functions related with symmetric conic domains.

## 1 Introduction

Suppose that the set of all analytic or holomorphic functions Ξ in the open unit disc U={ζ∈C:|ζ|<1} and that u∈Ξ has the series representation as

(1.1) $$u(\zeta )=\zeta +\sum_{m\geq 2}a_{m}\zeta^{m}.$$

We denote the basic subclass of analytic functions Ξ as

(1) $S=\left\{u\in \Xi :univalent\;and\;normalized\;by\;h(0)=0=h^{\prime }(0)-1;\zeta \in U\right\}$
(2) $N(\varrho )=\left\{u\in \Xi :ℜ\left(u^{\prime }(\zeta )\right)>\varrho ,0\leq \varrho <1;\zeta \in U\right\}$
(3) $S^{⁎}(\varrho )=\left\{u\in \Xi :ℜ\left(\frac{\zeta u^{\prime }(\zeta )}{u(\zeta )}\right)>\varrho ,0\leq \varrho <1;\zeta \in U\right\}$ starlike functions of order *ϱ*
  and
(4) $K(\varrho )=\left\{u\in \Xi :ℜ\left(\frac{{\left(\zeta u^{\prime }(\zeta )\right)}^{\prime }}{u^{\prime }}\right)>\varrho ,0\leq \varrho <1;\zeta \in U\right\}$ convex functions of order *ϱ*.

Let $u_{1}$ and $u_{2}$ be holomorphic function |w(ζ)|≤1,∀ζ⊂U and w(0)=0 with in **U** so that $u_{1}(\zeta )=u_{2}(w(\zeta ))$. We consider $u_{2}$ is subordinated by $u_{1}$ signified mathematically as $u_{1}≺u_{2}$. If $u_{2}$ is univalent, then $u_{1}≺u_{2}$ ⇔ $u_{1}(0)=u_{2}(0)$ and $u_{1}(U)\subseteq u_{2}(U)$.

For two holomorphic function

$$u_{1}(\zeta )=\sum_{m\geq 0}a_{m}\zeta^{m}\;\text{and}\;u_{2}(\zeta )=\sum_{m\geq 0}b_{m}\zeta^{m},\zeta \in U$$

the Hadamard (convolution) product $u_{1}(\zeta )⁎u_{2}(\zeta )$ is assumed as

$$u_{1}(\zeta )⁎u_{2}(\zeta )=\sum_{m\geq 0}a_{m}b_{m}\zeta^{m}.$$

We include a some notation of *q*-calculus exploited from the article [16], [17]. The *q*-analogue of *m* as

$${\left[m\right]}_{q}=\frac{1-q^{m}}{1-q}$$

and

$$m=\lim_{q\to 1}\frac{1-q^{m}}{1-q}.$$

The *q*-factorial, as below

$${\left[m\right]}_{q}!=\left\{ \begin{array}{c} {\left[m\right]}_{q}{\left[m-1\right]}_{q}\cdots {\left[1\right]}_{q},m=1,2,\cdots \\ 1\;\;m=0. \end{array}\right.$$

Moreover the *q*-derivative operator of u∈Ξ as given as

$$D_{q}u(\zeta )=\frac{h(q\zeta )-u(\zeta )}{\zeta (q-1)},\zeta \in U.$$

Consider

$$D_{q}\zeta^{m}={\left[m\right]}_{q}\zeta^{m-1}$$

and

$$D_{q}\left\{\sum_{m\geq 1}a_{m}\zeta^{m}\right\}=\sum_{m\geq 1}{\left[m\right]}_{q}a_{m}\zeta^{m-1},\;m\in N;\zeta \in U.$$

Jackson first used the idea of *q*-calculus [16], [17]. With remarkable way, he first proposed the *q*-integral and the well-known *q*-derivative. Subsequently, the geometric features of *q*-analysis have been mostly examined and discussed in terms of quantum groups [13], with a notable beginning in the early 1980s. In [3], [4], [5], the *q*-analogue of the well-known Baskakov Durrmeyer operator was presented based on the *q*-beta function. Two other important *q*-speculations regarding complex operators are the *q*-Picard integral operator and the *q*-Gauss-Weierstrass integral operator (see [6], [9], [14], [29]). The geometric features of these operators were scrutinized in detail. As demonstrated by [1], a number of operators are now being studied in [19], [24], [40] provides an explanation of the *q*-symmetric derivative operator and its numerous uses.

Numerous intriguing properties and characteristics of the Hurwitz-Lerch Zeta function Φ(z,s,b) have been revealed by recent investigations by Kiryakova [25], Lin and Srivastava [26], Choi et al. [8], Garg et al. [12], Ferreira and Lopez [10], Lin et al. [27] and others. Additionally, in 2007 by Srivastava [45], Srivastava and Attiya [44] (also see Raducanu and Srivastava [36], and Prajapat and Goyal [35]) as well as the references cited therein, have investigated and studied various subclasses of Ξ.

The following we recall a general *q*− analogue of Hurwitz-Lerch Zeta function (q-HLZ), $\Phi_{q}(\varrho ,\kappa ,\iota_{2})$ defined in [39], given by

$$\Phi_{q}(\zeta ,\kappa ,\varkappa ):=\sum_{m\geq 0}\frac{\zeta^{m}}{{\left[m+\varkappa \right]}_{q}^{\kappa }}$$

where

$$\varkappa \in C\setminus \{Z_{0}^{-}\};s\in C,R(\kappa )>1\;\;\;|\zeta |=1$$

and as usual, $Z_{0}^{-}:=Z\setminus \{N\}$, (Z:={±1,±2,±3,...});N:={1,2,3,...}.

Recently in [31], [39]introduced and discussed the linear operator:

$$J_{q}^{\kappa ,\varkappa }:\Xi \to \Xi$$

specified, with respect to the Hadamard product (or convolution), by

(1.2) $$J_{q}^{\kappa ,\varkappa }u(\zeta )=G_{\kappa ,\varkappa }⁎u(\zeta )$$

where,

(1.3) $$G_{q}^{\kappa ,\varkappa }(\zeta ):={\left[1+\varkappa \right]}_{q}^{\kappa }[\Phi_{q}(\zeta ,\kappa ,\varkappa )-{\left[\varkappa \right]}_{q}^{-\kappa }]\;\;\;\;(\zeta \in U)$$

and $\zeta \in U;\varkappa \in C\setminus \{Z_{0}^{-}\};\varpi \in C;u\in \Xi$ for different perspective of study on analytic and harmonic functions.

For **u** of the form (1.1), it is simple to note from (1.2) and (1.3) that we have

(1.4) $${ϒ}_{q}^{\kappa ,\varkappa }u(\zeta )=\zeta +\sum_{m\geq 2}L_{q}^{\kappa }(m,\varkappa )a_{m}\zeta^{m}=H(\zeta )$$

where

(1.5) $$L_{q}^{\kappa }(m,\varkappa )={\left(\frac{{\left[1+\varkappa \right]}_{q}}{{\left[m+\varkappa \right]}_{q}}\right)}^{\kappa }$$

(and throughout this paper unless otherwise mentioned) the parameters *ϰ* are constrained as follows:

$$\varkappa \in C\setminus \{Z_{0}^{-}\};\kappa \in C.$$

For u∈Ξ and ζ∈U

$$H(\zeta )=\zeta +\sum_{m\geq 2}{\left(\frac{{\left[1+\varkappa \right]}_{q}}{{\left[m+\varkappa \right]}_{q}}\right)}^{\kappa }a_{m}\zeta^{m}$$

and various choices of *κ* the *q*-HLZ includes the integral operators as listed below (see also [34], [44], [45].

Remark 1.1 $${ϒ}_{q}^{0,\varkappa }u(\zeta ):=u(\zeta ),{ϒ}_{q}^{1,\varkappa }u(\zeta ):=\int_{0}^{\zeta }\frac{u(t)}{t}dt:=A[u(\zeta )],\;(q-Alexander\;operator).{ϒ}_{q}^{1,1}u(\zeta ):=\frac{{\left[2\right]}_{q}}{\varrho }\int_{0}^{\zeta }\frac{u(t)}{t}dt:=A[u(\zeta )]\;(q-Libera\;operator).{ϒ}_{q}^{1,\varkappa }u(\zeta ):=\frac{{\left[1+\varkappa \right]}_{q}}{\zeta^{\varkappa }}\int_{0}^{\zeta }t^{1-\varkappa }u(t)dt:=F_{\varkappa }[u(\zeta )],(\varkappa >-1)(q-Bernardi\;operator).{ϒ}_{q}^{\kappa ,1}(u(\zeta ):=\zeta +\sum_{m\geq 2}{\left(\frac{{\left[2\right]}_{q}}{{\left[m+1\right]}_{q}}\right)}^{\kappa }a_{m}\zeta^{m}=I^{\kappa }[u(\zeta )],(\kappa >0),$$

note that if $q\to 1^{-}$, it closely related to some multiplier transformation studied by Fleet [11].

Special functions are extremely important in many areas of applied mathematics and sciences. Numerous researchers have studied the geometric properties of very unique special functions, as several studies have demonstrated (see [2], [33], [41]). After a thorough examination of relevant literature, it was discovered that the Ruscheweyh derivative operator [38], which is a differential operator that is well-known and frequently quoted, first appeared in the publication [42], [43], [46], [47], [48], [49], [50].

For  we denote by  and by  the class of *Janowski starlike functions* and *Janowski convex functions*, defined by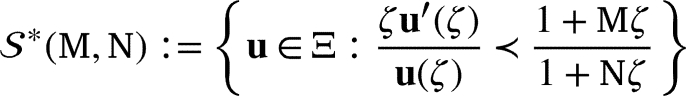 and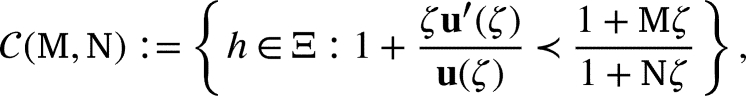 respectively (See [18] for a thorough analysis of the Janowski function class). Denote by P the family of functions $p(\zeta )=1+p_{1}\zeta +p_{2}\zeta^{2}+\cdots$ analytic in the **U** and  if and only if
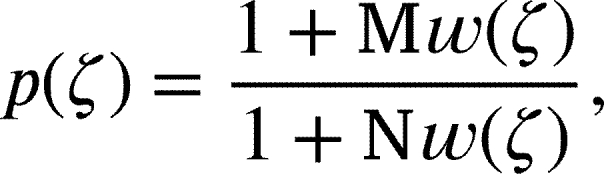
 where  and w(ζ) is the Schwarz function. Geometrically,  if and only if p(0)=1 and p(U) lies inside an open disc centered with center  on the real axis having radius  with diameter end points . On observing that $w(\zeta )=\frac{p(\zeta )-1}{p(\zeta )+1}$ for p∈P, we have  if and only if for some p∈P(1.6)
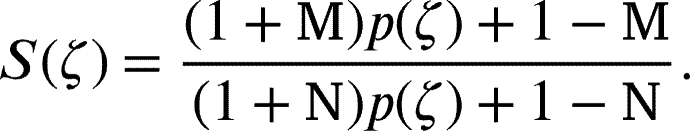


The conic domain $D_{k}=\left\{u+iv:\;u>k\sqrt{{\left(u-1\right)}^{2}+v^{2}}\right\}$, ${\overset{ˆ}{p}}_{℘,\sigma }(\zeta )$ plays the role of an extremal functions and is given by

(1.7) $${\overset{˜}{p}}_{k}(\zeta )=\left\{ \begin{array}{c} \frac{1+\zeta }{1-\zeta },\;k=0,\\ 1+\frac{2}{\pi^{2}}{\left(\log\frac{1+\sqrt{\zeta }}{1-\sqrt{\zeta }}\right)}^{2},\;k=1,\\ 1+\frac{2}{1-k^{2}}\sinh^{2}\left[\left(\frac{2}{\pi }\cos^{-1}k\right)\tan^{-1}h\sqrt{\zeta }\right],\;0<k<1,\\ 1+\frac{1}{k^{2}-1}\sin\left(\frac{\pi }{2Q(s)}\int_{0}^{\frac{u(\zeta )}{\sqrt{s}}}\frac{1}{\sqrt{1-x^{2}}\sqrt{1-{\left(sx\right)}^{2}}}dx\right)+\frac{1}{k^{2}-1},k>1. \end{array}\right.$$

where $u(\zeta )=\frac{\zeta -\sqrt{t}}{1-\sqrt{t\zeta }},\;t\in (0,\;1)$ and *t* is chosen such that $k=\cosh\left(\frac{\pi R^{\prime }(t)}{4R(t)}\right)$, with R(t) is Legendre's complete elliptic integral of the first kind and $R^{\prime }(t)$ is complementary integral of R(t). According to [21], [22], the function ${\overset{˜}{p}}_{k}(\zeta )$ provides the picture of **U** as a conic region that expresses symmetrically about the horizontal axis. Based on the equation ${\overset{˜}{p}}_{k}(\zeta )=1+\delta_{k}(\zeta )+\cdots$, [20] shows that, using (1.7), one may have

$$\delta_{k}=\left\{ \begin{array}{c} \frac{8{\left(\cos^{-1}k\right)}^{2}}{\pi^{2}(1-k^{2})},\;\;0\leq k<1,\\ \frac{8}{\pi^{2}},\;\;k=1,\\ \frac{\pi^{2}}{4(k^{2}-1)Q^{2}(s)\sqrt{s}(1+s)},\;\;k>1. \end{array}\right.$$

Motivated by aforementioned works on the study of conic regions impacted by Janowski function involving *q*-derivative was dealt in detail by Srivastava et al. [42], [43], [46], [47], [48], [49], [50] also see [28], [7], [30], [31], [32], [41], [52], [53] and references cited therein we defined a broad class of functions related to the *q*-Hurwitz-Lerch Zeta (*q*-HLZ) function, which is connected to the symmetric conic area that Janowski functions [23], [15] describe and denote by  as in below Definition 1.2 and discussed certain characteristic properties.

Definition 1.2 For u(ζ)∈Ξ defined in , if and only if
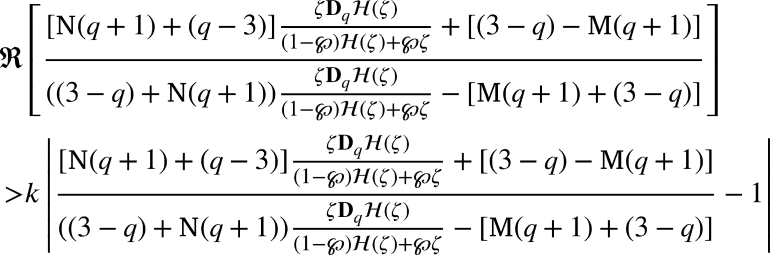
 or equivalently
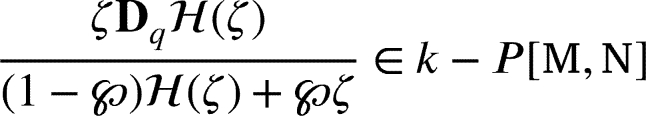
 where H(ζ) as in (1.4).

The new class that has not yet been explored is based on the (*q*-HLZ) function connected to the symmetric conic area that Janowski functions as follows, with the fixed parameters ℘=1 (and ℘=0).

Definition 1.3 For u(ζ)∈Ξ we let a new class , if it holds
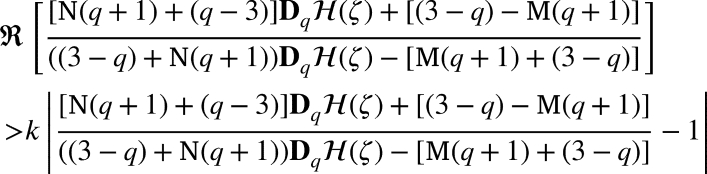
 or equivalently
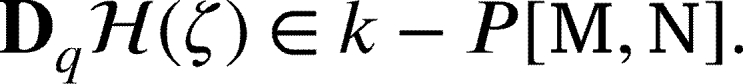

Definition 1.4 For u∈Ξ and , by fixing ℘=0, we deduce a new class , if it satify
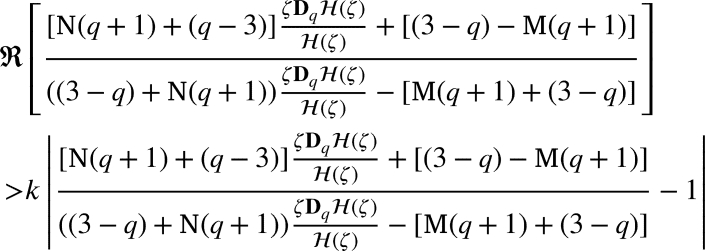
 or equivalently
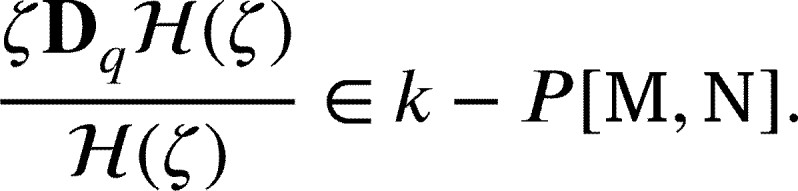

We find the well-known results, such as the coefficient bounds, partial sums results bounds and subordination results for this recently established function class in the following sections:

## 2 Coefficients bounds

To prove our result we recall the Rogosinski.

Lemma 2.1 *[37]**Let*$u(\zeta )=1+\sum_{m\geq 1}v_{m}\zeta^{m}$*be subordinate to*$H(\zeta )=1+\sum_{m\geq 1}V_{m}\zeta^{m}$*is univalent in***U***, then*

(2.1) $$|v_{m}|\leq |V_{1}|,\;m\geq 1.$$

In the following assertion, we prove a precondition for the functions that become part of .

Theorem 2.2 *A function*u∈Ξ*and has the form described in equation*(1.1)*will be class under**, provided that it fulfills the following condition*(2.2)
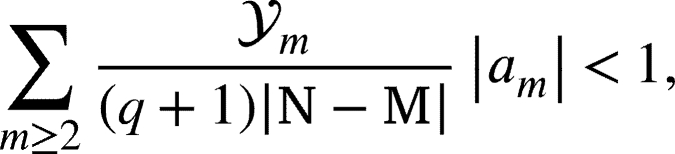
*where*(2.3)
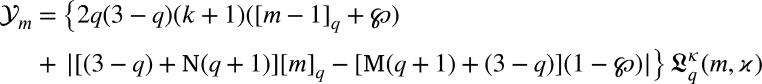
*where*$L_{q}^{\kappa }(m,\varkappa )$*as assumed in*(1.5)*.*

Proof Supposing that (2.2) holds true, it is sufficient to show that
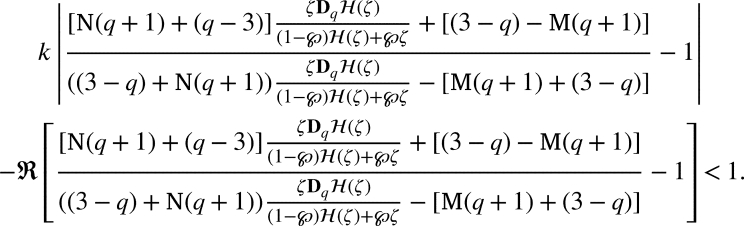
 For our convenient, let we write that
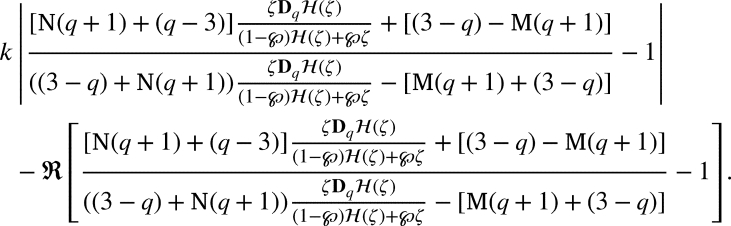

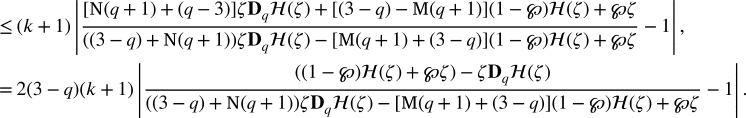
 Substituting for $D_{q}H(\zeta )$ and H(ζ) and upon simplification we get
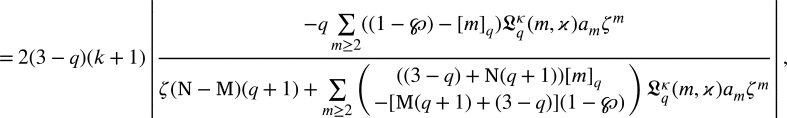

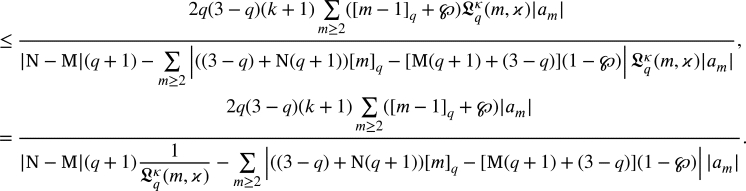
 If the above inequality bounded above by 1, we get
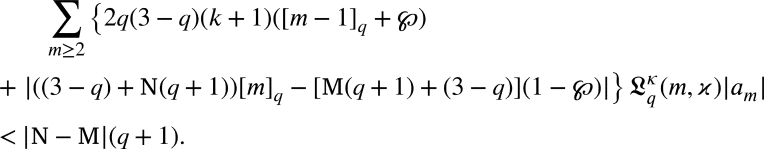
 Thus the proof is complete. □

Corollary 2.3 *For any* u∈Ξ *given in* (1.1) *and*  *, if it fulfills the criterion*

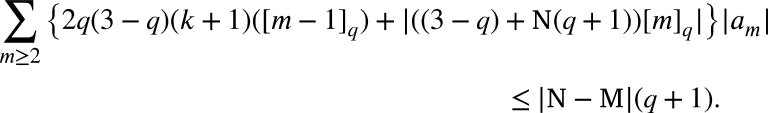

Theorem 2.4 *Let*  *,*  *as in* (1.1) *, then*
(2.4)
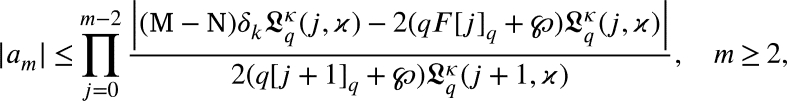

*where* $L_{q}^{\kappa }(m,\varkappa )$ *is defined in* (1.5) *.*

Proof By the definition of , we have

(2.5) $$\frac{\zeta D_{q}H(\zeta )}{(1-℘)H(\zeta )+℘\zeta }=p(\zeta ),$$

where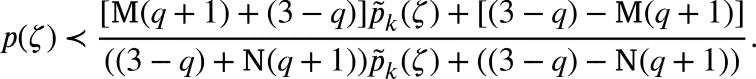 If ${\overset{˜}{p}}_{k}(\zeta )=1+\delta_{k}\zeta +\ldots$, then(2.6)
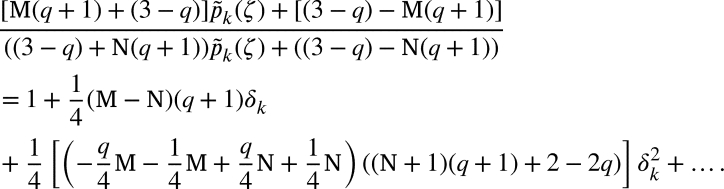
 Now, if $p(\zeta )=1+\sum_{m\geq 1}p^{m}\zeta^{m}$, then by (2.1) and (2.6), we arrived(2.7)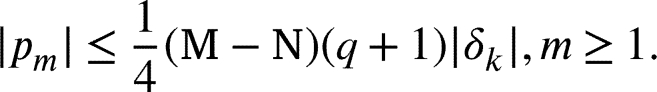 Now, from (2.5), we arrived

$$\zeta D_{q}H(\zeta )=p(\zeta )\left((1-℘)H(\zeta )+℘\zeta \right)$$

and using $p(\zeta )=1+\sum_{m\geq 1}p_{m}\zeta^{m}$ with $p_{0}=1$, we arrived

$$\zeta +\sum_{m\geq 2}{\left[m\right]}_{q}L_{q}^{\kappa }(m,\varkappa )a_{m}\zeta^{m}=\left(1+\sum_{m\geq 1}p_{m}\zeta^{m}\right)\left((1-℘)\left(\zeta +\sum_{m\geq 2}L_{q}^{\kappa }(m,\varkappa )a_{m}\zeta^{m}\right)+℘\zeta \right)$$

by simple computation we have

$$\sum_{m\geq 1}\left({\left[m\right]}_{q}-(1-℘)\right)L_{q}^{\kappa }(m,\varkappa )a_{m}\zeta^{m}=\left(\sum_{m\geq 1}p_{m}\zeta^{m}\right)\left((1-℘)\sum_{m\geq 1}L_{q}^{\kappa }(m,\varkappa )a_{m}\zeta^{m}\right).$$

Using the Cauchy product, we arrived

$$\sum_{m\geq 1}(q{\left[m-1\right]}_{q}+℘)L_{q}^{\kappa }(m,\varkappa )a_{m}\zeta^{m}=(1-℘)\sum_{m\geq 1}\sum_{j=1}^{m-1}L_{q}^{\kappa }(j,\varkappa )a_{j}p_{m-j}\zeta^{m}.$$

Equating like coefficients of $\zeta^{m}$, we have

$$(q{\left[m-1\right]}_{q}+℘)L_{q}^{\kappa }(m,\varkappa )a_{m}=(1-℘)\sum_{j=1}^{m-1}L_{q}^{\kappa }(j,\varkappa )a_{j}p_{m-j},$$

which yields

$$a_{m}=\frac{\left(1-℘\right)}{\left(q{\left[m-1\right]}_{q}+℘)L_{q}^{\kappa }(m,\varkappa \right)}\sum_{j=1}^{m-1}L_{q}^{\kappa }(j,\varkappa )a_{j}p_{m-j}.$$

By (2.7), we have(2.8)
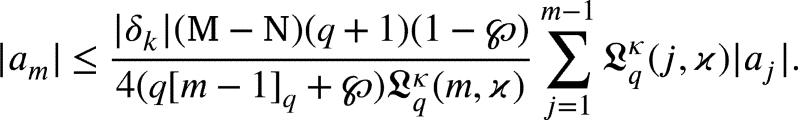
 Now, we prove that
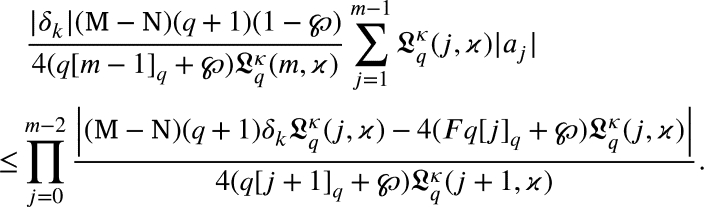
 By the induction method we can proceed to this proof. For m=2, from (2.8), we arrived
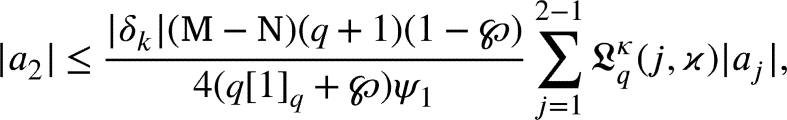
 it becomes to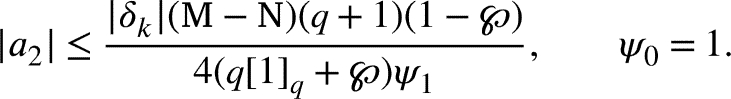 From (2.4)
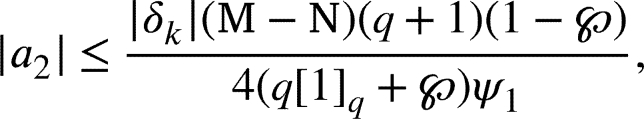
 substituting m=3, from (2.8), we arrived
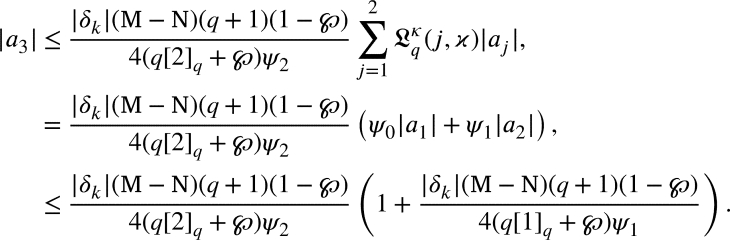
 From (2.4), we have
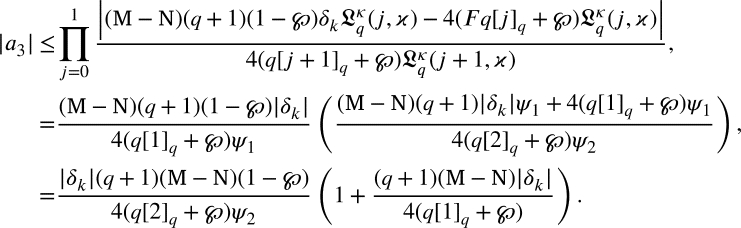
 Suppose that for m=m+1 the hypothesis holds. From (2.8), we get
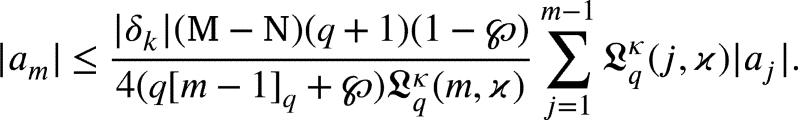
 From (2.4), we get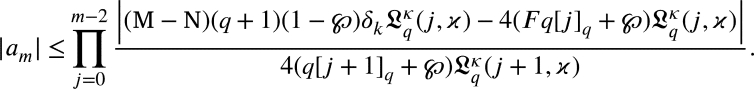 Using induction principle
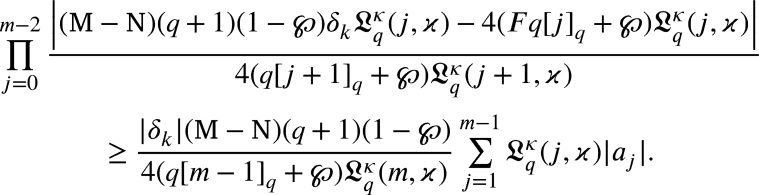
 Multiplying both the sides by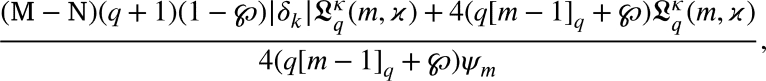 we arrived
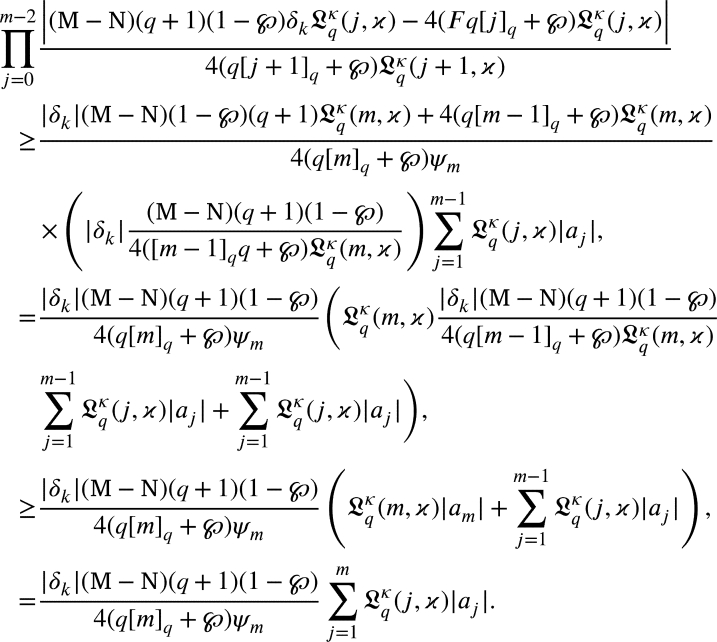
 That is,
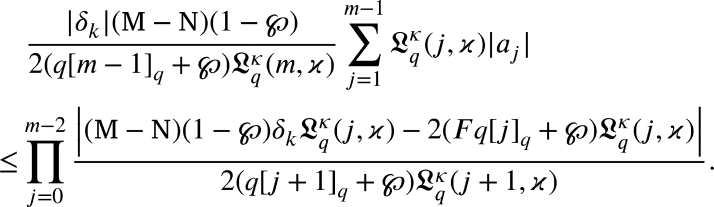
 Thus, the outcome is true for m=m+1. Consequently, the induction principle concludes that for any m≥2., (2.4) is true. □

## 3 Bounds of the partial sums

After reviewing the findings of Silvia [40] and Silverman [41], we examine how a function represented by (1.1) may be divided into its component parts using a sequence of cumulative partial terms

$$u_{1}(\zeta )=\zeta$$

and

$$\sum_{j=2}^{m}a_{j}\zeta^{j}=u_{m}(\zeta ).$$

Numerous authors have studied partial sums for various subclasses (see [30], [32], [39], [46], [47], [48] and references therein). For the functions u(ζ)∈Ξ of the class , we analyze the lower bounds for $\frac{u(\zeta )}{u_{m}{\left(\zeta \right)}^{\prime }}$, $\frac{h^{\prime }(\zeta )}{u_{m}^{\prime }{\left(\zeta \right)}^{\prime }}$, $\frac{u_{m}(\zeta )}{u(\zeta )}$ and $\frac{u_{m}^{\prime }(\zeta )}{u(\zeta )}$.

Theorem 3.1 *For any**, then*

$$ℜ\left\{\frac{u(\zeta )}{u_{m}(\zeta )}\right\}\geq 1-\frac{ℵ}{Y_{m+1}},$$

*where*$Y_{m+1}$*is stated by*(2.3)*and**. The function*

(3.1) $$u(\zeta )=\zeta +\frac{ℵ}{Y_{m+1}}\zeta^{m+1},$$

*provides the sharp result.*

Proof Assume that

$$w(\zeta )=\frac{Y_{m+1}}{ℵ}\left[\frac{u(\zeta )}{u_{m}(\zeta )}-\left(1-\frac{ℵ}{Y_{m+1}}\right)\right],=\frac{Y_{m+1}}{ℵ}\frac{u(\zeta )}{u_{m}(\zeta )}-\frac{Y_{m+1}}{ℵ}+1,$$

which reduces to

$$w(\zeta )=\frac{Y_{m+1}\left(1+\sum_{j=2}^{\infty }a_{j}\zeta^{j-1}\right)}{ℵ\left(1+\sum_{j=2}^{m}a_{j}\zeta^{j-1}\right)}-\frac{Y_{m+1}}{ℵ}+1,=\frac{1+\sum_{j=2}^{m}a_{j}\zeta^{j-1}+\frac{Y_{m+1}}{ℵ}\sum_{j=m+1}^{\infty }a_{j}\zeta^{j-1}}{1+\sum_{j=2}^{m}a_{j}\zeta^{j-1}}.$$

Using this, one may have

$$\left|\frac{w(\zeta )-1}{w(\zeta )+1}\right|\leq \frac{\frac{Y_{m+1}}{ℵ}\sum_{j=m+1}^{\infty }|a_{j}|}{2-2\sum_{j=2}^{m}|a_{j}|-\frac{Y_{m+1}}{ℵ}\sum_{j=m+1}^{\infty }|a_{j}|}.$$

Now,

$$\left|\frac{w(\zeta )-1}{w(\zeta )+1}\right|\leq 1,$$

if

(3.2) $$\sum_{j=2}^{m}|a_{j}|+\frac{Y_{m+1}}{ℵ}\sum_{j=m+1}^{\infty }|a_{j}|\leq 1.$$

It suffices to prove that the upper bound of $\sum_{j=2}^{\infty }\frac{Y_{j}}{ℵ}|a_{j}|$, on the left side of (3.2), if

$$\sum_{j=2}^{m}|a_{j}|+\frac{Y_{m+1}}{ℵ}\sum_{j=m+1}^{\infty }|a_{j}|\leq \sum_{j=2}^{\infty }\frac{Y_{j}}{ℵ}|a_{j}|,$$

which leads to the expression

$$\sum_{j=2}^{m}\frac{Y_{j}-ℵ}{ℵ}|a_{j}|+\sum_{j=m+1}^{\infty }\left(\frac{Y_{j}-Y_{m+1}}{ℵ}\right)|a_{j}|\geq 0.$$

To verify the sharpness, of (3.1) when $\zeta =re^{\frac{i\pi }{m}}$.

$$\frac{u(\zeta )}{u_{m}(\zeta )}=1+\frac{ℵ}{Y_{m+1}}\zeta^{m},=1+\frac{ℵ}{Y_{m+1}}r^{m}e^{i\pi },=1-\frac{ℵr^{m}}{Y_{m+1}},=\frac{Y_{m+1}-ℵ}{Y_{m+1}}\;when\;r\to 1.$$

 □

Theorem 3.2 *For any*  *, then*

(3.3) $$ℜ\left\{\frac{u_{m}(\zeta )}{u(\zeta )}\right\}\geq \frac{Y_{m+1}}{Y_{m+1}+ℵ},$$

*where* $Y_{m+1}$ *is as in* (2.3) *and*  *. The bound* (3.3) *is sharp for function given in* (3.1) *.*

Proof Again let us assume

$$w(\zeta )=\frac{Y_{m+1}+ℵ}{ℵ}\left[\frac{u_{m}(\zeta )}{u(\zeta )}-\frac{Y_{m+1}}{Y_{m+1}+ℵ}\right],=\frac{\left(Y_{m+1}+ℵ)u_{m}(\zeta \right)}{ℵu(\zeta )}-\frac{Y_{m+1}}{ℵ}.$$

Now we get

$$w(\zeta )=\frac{(Y_{m+1}+ℵ)\left(1+\sum_{j=2}^{m}a_{j}\zeta^{j-1}\right)}{ℵ\left(1+\sum_{j=2}^{\infty }a_{j}\zeta^{j-1}\right)}-\frac{Y_{m+1}}{ℵ},=\frac{1+\sum_{j=2}^{m}a_{j}\zeta^{j-1}-\frac{Y_{m+1}}{ℵ}\sum_{j=m+1}^{\infty }a_{j}\zeta^{j-1}}{1+\sum_{j=2}^{\infty }a_{j}\zeta^{j-1}}.$$

Thus we have

$$\frac{w(\zeta )-1}{w(\zeta )+1}=\frac{-\left(1+\frac{Y_{m+1}}{ℵ}\right)\sum_{j=m+1}^{\infty }a_{j}\zeta^{j-1}}{2+2\sum_{j=2}^{m}a_{j}\zeta^{j-1}+\left(1-\frac{Y_{m+1}}{ℵ}\right)\sum_{j=m+1}^{\infty }a_{j}\zeta^{j-1}},$$

which simplifies that

$$\left|\frac{w(\zeta )-1}{w(\zeta )+1}\right|\leq \frac{\left(1+\frac{Y_{m+1}}{ℵ}\right)\sum_{j=m+1}^{\infty }|a_{j}|}{2-2\sum_{j=2}^{m}|a_{j}|-\left(1-\frac{Y_{m+1}}{ℵ}\right)\sum_{j=m+1}^{\infty }|a_{j}|}.$$

Now,

$$\left|\frac{w(\zeta )-1}{w(\zeta )+1}\right|\leq 1,$$

if

(3.4) $$\sum_{j=2}^{m}|a_{j}|+\sum_{j=m+1}^{\infty }|a_{j}|\leq 1.$$

Proving the upper bound of $\sum_{j=2}^{\infty }\frac{Y_{j}}{ℵ}|a_{j}|$, on the left side of (3.4) is appropriate if

$$\sum_{j=2}^{m}|a_{j}|+\sum_{j=m+1}^{\infty }|a_{j}|\leq \sum_{j=2}^{\infty }\frac{Y_{j}}{ℵ}|a_{j}|,$$

which tends to

$$\sum_{j=2}^{m}\left(\frac{Y_{j}}{ℵ}-1\right)|a_{j}|+\sum_{j=m+1}^{\infty }\left(\frac{Y_{j}}{ℵ}-1\right)|a_{j}|\geq 0.$$

That is,

$$\sum_{j=2}^{\infty }\left(\frac{Y_{j}}{ℵ}-1\right)|a_{j}|\geq 0.$$

Hence, equality posses for u(ζ), as given in (3.1). □

Theorem 3.3 *For any*  *, then*

(3.5) $$ℜ\left\{\frac{h^{\prime }(\zeta )}{u_{m}^{\prime }(\zeta )}\right\}\geq 1-\frac{ℵ(m+1)}{Y_{m+1}},$$

*where* $Y_{m+1}$ *is as in* (2.3) *and*  *. The bound* (3.5) *is sharp for function given in* (3.1) *.*

Proof Let us take a function w(ζ),

$$w(\zeta )=\frac{Y_{m+1}}{ℵ(m+1)}\left[\frac{h^{\prime }(\zeta )}{u_{m}^{\prime }(\zeta )}-\frac{Y_{m+1}-ℵ(m+1)}{g(m+1)}\right],\text{which becomes}\;w(\zeta )=\frac{Y_{m+1}\left(1+\sum_{j=2}^{m}ja_{j}\zeta^{j-1}\right)}{ℵ(m+1)\left(1+\sum_{j=2}^{m}ja_{j}\zeta^{j-1}\right)}-\frac{Y_{m+1}-ℵ(m+1)}{g(m+1)},=\frac{1+\sum_{j=2}^{m}ja_{j}\zeta^{j-1}+\frac{Y_{m+1}}{ℵ(m+1)}\sum_{j=m+1}^{\infty }ja_{j}\zeta^{j-1}}{1+\sum_{j=2}^{m}ja_{j}\zeta^{j-1}}.$$

It reduces us to

$$\frac{w(\zeta )-1}{w(\zeta )+1}=\frac{\frac{Y_{m+1}}{ℵ(m+1)}\sum_{j=m+1}^{\infty }ja_{j}\zeta^{j-1}}{2+2\sum_{j=2}^{m}ja_{j}\zeta^{j-1}+\frac{Y_{m+1}}{ℵ(m+1)}\sum_{j=m+1}^{\infty }ja_{j}\zeta^{j-1}},$$

which tends to

$$\left|\frac{w(\zeta )-1}{w(\zeta )+1}\right|\leq \frac{\frac{Y_{m+1}}{ℵ(m+1)}\sum_{j=m+1}^{\infty }j|a_{j}|}{2-2\sum_{j=2}^{m}j|a_{j}|-\frac{Y_{m+1}}{ℵ(m+1)}\sum_{j=m+1}^{\infty }j|a_{j}|}.$$

Now,

$$\left|\frac{w(\zeta )-1}{w(\zeta )+1}\right|\leq 1,$$

if

(3.6) $$\sum_{j=2}^{m}j|a_{j}|+\frac{Y_{m+1}}{ℵ(m+1)}\sum_{j=m+1}^{\infty }j|a_{j}|\leq 1.$$

It is adequate to prove that the upper bound of $\sum_{j=2}^{\infty }\frac{Y_{j}}{ℵ}|a_{j}|$, on the left side of (3.6), if

$$\sum_{j=2}^{m}j|a_{j}|+\frac{Y_{m+1}}{ℵ(m+1)}\sum_{j=m+1}^{\infty }j|a_{j}|\leq \sum_{j=2}^{\infty }\frac{Y_{j}}{ℵ}|a_{j}|,$$

which tends to the below form

$$\sum_{j=2}^{m}\left(\frac{Y_{j}}{ℵ}-j\right)|a_{j}|+\sum_{j=m+1}^{\infty }\left(\frac{Y_{j}}{ℵ}-\frac{jY_{m+1}}{ℵ(m+1)}\right)|a_{j}|\geq 0.$$

 □

Theorem 3.4 *If*  *, then*

(3.7) $$ℜ\left\{\frac{u_{m}^{\prime }(\zeta )}{h^{\prime }(\zeta )}\right\}\geq \frac{Y_{m+1}}{Y_{m+1}+ℵ(m+1)},$$

*where* $Y_{m+1}$ *is as in* (2.3) *and*  *. The result* (3.7) *is sharp for function given in* (3.1) *.*

Proof Let us consider a function w(ζ) as below

$$w(\zeta )=\frac{Y_{m+1}+ℵ(m+1)}{ℵ(m+1)}\left[\frac{u_{m}^{\prime }(\zeta )}{h^{\prime }(\zeta )}-\frac{Y_{m+1}}{Y_{m+1}+ℵ(m+1)}\right]$$

that is,

$$w(\zeta )=\frac{(Y_{m+1}+ℵ(m+1))\left(1+\sum_{j=2}^{\infty }ja_{j}\zeta^{j-1}\right)}{ℵ(m+1)\left(1+\sum_{j=2}^{m}ja_{j}\zeta^{j-1}\right)}-\frac{Y_{m+1}}{ℵ(m+1)},=\frac{1+\sum_{j=2}^{m}ja_{j}\zeta^{j-1}-\frac{Y_{m+1}}{ℵ(m+1)}\sum_{j=m+1}^{\infty }ja_{j}\zeta^{j-1}}{1+\sum_{j=2}^{\infty }ja_{j}\zeta^{j-1}}.$$

This yield us to

$$\frac{w(\zeta )-1}{w(\zeta )+1}=\frac{\sum_{j=2}^{m}ja_{j}\zeta^{j-1}-\sum_{j=2}^{\infty }ja_{j}\zeta^{j-1}-\frac{Y_{m+1}}{ℵ(m+1)}\sum_{j=m+1}^{\infty }ja_{j}\zeta^{j-1}}{2+\sum_{j=2}^{m}ja_{j}\zeta^{j-1}+\sum_{j=2}^{\infty }ja_{j}\zeta^{j-1}-\frac{Y_{m+1}}{ℵ(m+1)}\sum_{j=m+1}^{\infty }ja_{j}\zeta^{j-1}},=\frac{-\sum_{j=m+1}^{\infty }\left(1+\frac{Y_{m+1}}{ℵ(m+1)}\right)ja_{j}\zeta^{j-1}}{2+2\sum_{j=2}^{m}ja_{j}\zeta^{j-1}+\sum_{j=m+1}^{\infty }\left(1-\frac{Y_{m+1}}{ℵ(m+1)}\right)ja_{j}\zeta^{j-1}}.$$

That is

$$\left|\frac{w(\zeta )-1}{w(\zeta )+1}\right|\leq \frac{\left(1+\frac{Y_{m+1}}{ℵ(m+1)}\right)\sum_{j=m+1}^{\infty }j|a_{j}|}{2-2\sum_{j=2}^{m}j|a_{j}|-\left(1-\frac{Y_{m+1}}{ℵ(m+1)}\right)\sum_{j=m+1}^{\infty }j|a_{j}|}.$$

Now,

$$\left|\frac{w(\zeta )-1}{w(\zeta )+1}\right|\leq 1,$$

if

(3.8) $$\sum_{j=2}^{m}j|a_{j}|+\sum_{j=m+1}^{\infty }j|a_{j}|\leq 1.$$

The left part of (3.8) as bounded above by $\sum_{j=2}^{\infty }\frac{Y_{j}}{ℵ}|a_{j}|$, if

$$\sum_{j=2}^{m}j|a_{j}|+\sum_{j=m+1}^{\infty }j|a_{j}|\leq \sum_{j=2}^{\infty }\frac{Y_{j}}{ℵ}|a_{j}|,$$

which reduced as

$$\sum_{j=2}^{m}j|a_{j}|+\sum_{j=m+1}^{\infty }j|a_{j}|\leq \sum_{j=2}^{m}\frac{Y_{j}}{ℵ}|a_{j}|+\sum_{j=m+1}^{\infty }\frac{Y_{j}}{ℵ}|a_{j}|,$$

thus we have

$$\sum_{j=2}^{m}\left(\frac{Y_{j}}{ℵ}-j\right)|a_{j}|+\sum_{j=m+1}^{\infty }\left(\frac{Y_{j}}{ℵ}-j\right)|a_{j}|\geq 0.$$

Therefore,

$$\sum_{j=2}^{\infty }\left(\frac{Y_{j}}{ℵ}-j\right)|a_{j}|\geq 0.$$

 □

## 4 Subordination results

Now due to Wilf [51], we state subordinating factor sequence which are more essential for our discussion.

Definition 4.1 *(Subordinating Factor Sequence)**[51]*: A sequence ${\left\{b_{m}\right\}}_{n=1}^{\infty }$ of complex numbers is said to be a subordinating sequence if, u∈Ξ given by (1.1) is holomorphic, univalent and convex in **U**, then

$$\sum_{m\geq 1}b_{m}a_{m}\zeta^{m}≺h(\zeta ),\;\;\;\;\zeta \in U.$$

Lemma 4.2 *The sequence*${\left\{b_{n}\right\}}_{n=1}^{\infty }$*is a subordinating factor sequence if and only if*

$$\text{Re}\;\left\{1+2\sum_{n=1}^{\infty }b_{m}\zeta^{m}\right\}>0,\;\;\;\;\zeta \in U.$$

Theorem 4.3 *Let**and*g(ζ)∈K*then*

(4.1) $$\frac{Y_{2}(\varkappa ,℘,q)}{2[1-\xi +Y_{2}(\varkappa ,℘,q)]}(h⁎g)(\zeta )≺g(\zeta )$$

*where*
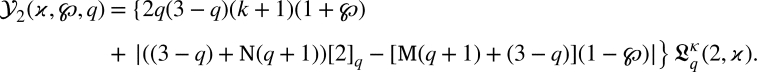
*and*$L_{q}^{\kappa }(2,\varkappa )={\left(\frac{{\left[1+\varkappa \right]}_{q}}{{\left[2+\varkappa \right]}_{q}}\right)}^{\kappa }$*is from*(1.5)*.*

(4.2) $$\text{Re}\;\left\{h(\zeta )\right\}>-\frac{\left[1-\xi +Y_{2}(\varkappa ,℘,q)\right]}{Y_{2}(\varkappa ,℘,q)},\;\;\;\;\zeta \in U.$$

*The constant factor*$\frac{Y_{2}(\varkappa ,℘,q)}{2[1-\xi +Y_{2}(\varkappa ,℘,q)]}$*in*(4.1)*cannot be substituted by a greater number.*

Proof Since  and assume that $g(\zeta )=\zeta +\sum_{n=2}^{\infty }b_{m}\zeta^{m}\in K$. Then

$$\frac{Y_{2}(\varkappa ,℘,q)}{2[1-\xi +Y_{2}(\varkappa ,℘,q)]}(h⁎g)(\zeta )\;\;\;\;\;\;\;\;\;\;\;\;\;\;\;\;\;\;\;\;\;\;\;\;\;\;\;=\frac{Y_{2}(\varkappa ,℘,q)}{2[1-\xi +Y_{2}(\varkappa ,℘,q)]}\left(\zeta +\sum_{m\geq 2}b_{m}a_{m}\zeta^{m}\right).$$

Therefore, by Definition 4.1, the subordination result holds if

$${\left\{\frac{Y_{2}(\varkappa ,℘,q)}{2[1-\xi +Y_{2}(\varkappa ,℘,q)]}\right\}}_{m=1}^{\infty }$$

is a subordinating factor sequence, with $a_{1}=1$. In sight of Lemma 4.2, this is equal to the subsequent inequality

(4.3) $$\text{Re }\left\{1+\sum_{m\geq 1}\frac{Y_{2}(\varkappa ,℘,q)}{\left[1-\xi +Y_{2}(\varkappa ,℘,q)\right]}a_{m}\zeta^{m}\right\}>0,\;\;\;\;\zeta \in U.$$

For n≥2 we note that $\frac{Y_{n}(\varkappa ,℘,q)}{1-\xi }$ is increasing function and in particular

$$\frac{Y_{2}(\varkappa ,℘,q)}{1-\xi }\leq \frac{Y_{m}(\varkappa ,℘,q)}{1-\xi },\;\;\;\;m\geq 2,$$

therefore, for |ζ|=r<1, we have

$$\text{Re }\left\{1+\frac{Y_{2}(\varkappa ,℘,q)}{\left[1-\xi +Y_{2}(\varkappa ,℘,q)\right]}\sum_{m\geq 1}a_{m}\zeta^{m}\right\}=\text{Re }\left\{1+\frac{Y_{2}(\varkappa ,℘,q)}{\left[1-\xi +Y_{2}(\varkappa ,℘,q)\right]}\zeta +\frac{\sum_{m\geq 2}Y_{2}(\varkappa ,℘,q)a_{m}\zeta^{m}}{\left[1-\xi +Y_{2}(\varkappa ,℘,q)\right]}\right\}\geq 1-\frac{Y_{2}(\varkappa ,℘,q)}{\left[1-\xi +Y_{2}(\varkappa ,℘,q)\right]}r-\frac{\sum_{m\geq 2}\left|Y_{m}(\varkappa ,℘,q)a_{m}\right|r^{m}}{\left[1-\xi +Y_{2}(\varkappa ,℘,q)\right]}\geq 1-\frac{Y_{2}(\varkappa ,℘,q)}{\left[1-\xi +Y_{2}(\varkappa ,℘,q)\right]}r-\frac{1-\xi }{\left[1-\xi +Y_{2}(\varkappa ,℘,q)\right]}r>0,\;\;\;\;|\zeta |=r<1,$$

by the assertion (2.2) of Theorem 2.2. This clearly proves (4.3) and hence (4.1).

By fixing

$$g(\zeta )=\frac{\zeta }{1-\zeta }=\zeta +\sum_{m\geq 2}\zeta^{m}\in K,$$

inequality (4.2) follows from (4.1). Subsequently we consider the function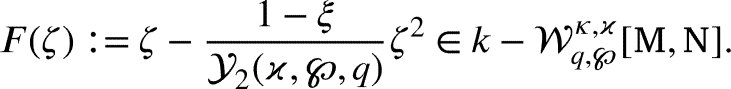 For this function (4.1) becomes

$$\frac{Y_{2}(\varkappa ,℘,q)}{2[1-\xi +Y_{2}(\varkappa ,℘,q)]}F(\zeta )≺\frac{\zeta }{1-\zeta }.$$

It is easily verified that

$$\min\left\{\text{Re }\left(\frac{Y_{2}(\varkappa ,℘,q)}{2[1-\xi +Y_{2}(\varkappa ,℘,q)]}F(\zeta )\right)\right\}=-\frac{1}{2},\;\;\;\;\zeta \in U.$$

This proves that the constant $\frac{Y_{2}(\varkappa ,℘,q)}{2[1-\xi +Y_{2}(\varkappa ,℘,q)]}$ cannot be substituted by a greater number. □

**Conclusion:** Our research introduces and investigates a novel family of analytic functions based on the convolution characterized by a *q*-HLZ function. Through rigorous analysis, we unveil several key findings, including coefficient inequalities and other noteworthy characteristics within this function class. Our approach allowed us to analyze the intriguing aspects of this subclass. Furthermore, we delve into the determination of partial sums, starlikeness radii, and coefficient estimates for the class of Janowski starlike functions in the context of symmetric conic domains. By fixing the values of parameter ℘ as mentioned in Definition 1.3, Definition 1.4 we can derive analogues results given in Theorem 2.2, Theorem 4.3. Moreover specializing the parameters as in Remark 1.1, new subclasses can be defined and discussed for the results given in Theorem 2.2, Theorem 4.3. This exploration not only contributes to the theoretical framework of analytical functions but also provides valuable insights into the properties and behavior of the *q*-HLZ function and its associated subclasses. Our findings not only advance the understanding of these mathematical structures but also pave the way for potential applications in diverse scientific and engineering disciplines moreover inspired to develop this concept to the classes of bi-univalent functions, meromorphic functions, etc.

## Ethical approval

This article does not contain any studies with human participants or animals performed by any of the authors.

## Funding statement

The research did not receive any funding.

## CRediT authorship contribution statement

**K. Uma:** Writing – original draft, Methodology, Investigation, Formal analysis. **K. Vijaya:** Writing – review & editing, Validation, Supervision, Methodology, Investigation, Conceptualization.

## Declaration of Competing Interest

The authors declare that they have no known competing financial interests or personal relationships that could have appeared to influence the work reported in this paper.

## Data Availability

No data were used in this paper.
